# Genome-Wide Association Study of Daughter Pregnancy Rate in Crossbred Dairy Cows

**DOI:** 10.3390/ijms262211149

**Published:** 2025-11-18

**Authors:** Ruifei Yang, Zuoxiang Liang, Dzianis Prakapenka, Li Ma, Yang Da

**Affiliations:** 1 Department of Animal Science, University of Minnesota, Saint Paul, MN 55108, USA; 2 College of Animal Science and Technology, Yunnan Agricultural University, Kunming 650201, China; 3 Department of Epidemiology and Health Statistics, School of Public Health, Fujian Medical University, Fuzhou 350108, China; 4 Department of Animal and Avian Sciences, University of Maryland, College Park, MD 20742, USA; lima@umd.edu

**Keywords:** GWAS, daughter pregnancy rate, heterosis, SNP, crossbred, cow

## Abstract

A genome-wide association study (GWAS) of daughter pregnancy rate (DPR) was conducted using 75,133 SNPs and 40,203 first lactation crossbred dairy cows mostly from Jersey–Holstein crosses. The GWAS analysis detected 6528 additive effects, 65 dominance effects, 1638 additive × additive (A × A) effects, 3 additive × dominance effects, and 18 intra-chromosome dominance × dominance (D × D) effects. Of the 1638 A × A effects, 1634 were intra-chromosome and four were inter-chromosome A × A effects. The distance between two SNPs with intra-chromosome epistasis effects was in the range of 3.61 Kb to 2.68 Mb, and many interacting SNP pairs were within the same genes. The additive and A × A effects were distributed on all chromosomes showing genome-wide involvement in DPR heterosis. The dominance and D × D effects all had homozygous advantages and heterozygous disadvantages. The GWAS results identified four genetic mechanisms underlying DPR heterosis in crossbred dairy cows: complementary additive effects from different breeds and new additive effects due to cross breeding, two-locus allelic interactions between loci and between breeds, within-locus allelic interactions between breeds, and genotype × genotype interactions enabled by allelic interactions between breeds. Results in this study provided a novel understanding about the genetic factors and mechanisms underlying DPR heterosis in crossbred dairy cows.

## 1. Introduction

Daughter pregnancy rate (DPR) is the percentage of cows that become pregnant during each 21-day period and is an important fertility trait in dairy cows [1]. The Holstein breed is the largest dairy breed in the U.S. with high production levels but low DPR, which was in decline since 1960s until about 2000 when a trend reversal occurred, but the declines since the 1960s were not fully recovered [2]. In contrast to purebred dairy cows, crossbred dairy cows typically have improved fertility and survival rate relative to pure breeds and these advantages are most striking relative to Holsteins [3,4,5,6]. The phenotypic advantage of crossbreds over the phenotypic performance of their parental breeds is termed as heterosis [7,8]. The previously reported DPR heterosis was also confirmed in this study (see Section 3). Several data-driven hypotheses about the genetic mechanism of heterosis have been proposed, including more superior alleles in hybrids than in parental lines [9]; heterozygous advantage where heterozygous genotypes outperformed homozygous genotypes; a genetic phenomenon termed as dominance [10] or overdominance [11,12]; the dominance model where the genotypes with one or two dominant alleles per locus were more favorable than the homozygous recessive genotypes [13]; and negative dominance and dominance × dominance epistasis effects [14]. These different hypotheses indicated that the genetic mechanism of heterosis could be different for different traits and species and that more studies on the genetic mechanism of heterosis are needed to build a consensus towards understanding the genetic mechanism of heterosis. The U.S. dairy genomic evaluation database has accumulated a large dairy crossbred population with DPR phenotypic observations and genotypic data of genome-wide single nucleotide polymorphism (SNP) markers. Utilizing these resources, this study aimed at identifying genetic mechanisms of DPR heterosis through a genome-wide association study (GWAS) of additive, dominance, and pairwise epistasis effects.

## 2. Results and Discussion

The GWAS analysis detected 6528 additive effects and 65 dominance effects with log_10_(1/p) > 8, and 1659 pairwise epistasis effects with log_10_(1/p) > 12 for DPR in the 40,203 crossbred dairy cows. Of the 1659 pairwise effects, 1638 were additive × additive (A × A) effects, 3 were additive × dominance (A × D) or dominance × additive (D × A) effects, and 18 were dominance × dominance (D × D) effects. Of the 1638 A × A effects, 1634 were intra-chromosome and four were inter-chromosome A × A effects. The only inter-chromosome pairwise epistasis effect was a D × A effect. Details of these effects are described below.

### 2.1. Additive Effects

**Figure 1:**
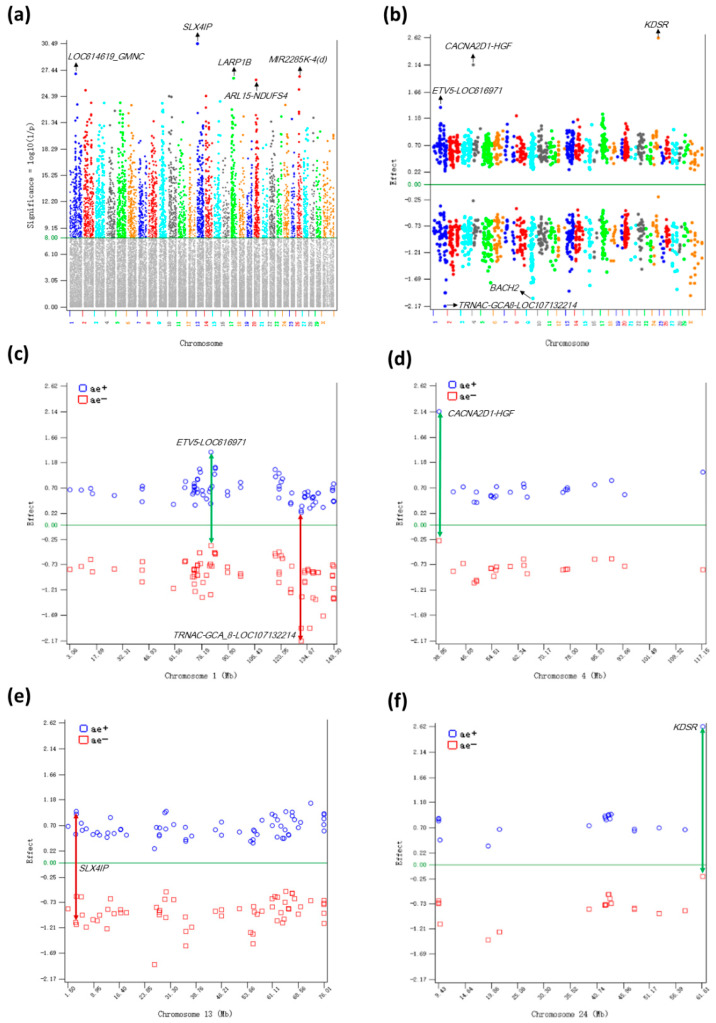
Additive effects of DPR. (**a**) Manhattan plot of genome-wide additive effects, showing genome-wide distribution of additive effects and the lack of a sharply significant chromosome region; (**b**) positive and negative allelic effects of the top 1000 most significant additive effects; (**c**) allelic effects of Chr01 among the top 1000 additive effects; (**d**) allelic effects of Chr04 among the top 1000 additive effects; (**e**) allelic effects of Chr13 among the top 1000 additive effects; (**f**) allelic effects of Chr24 among the top 1000 additive effects. ‘ae+’ is the allelic effect of the positive allele. ‘ae-’ is the allelic effect of the negative allele. Green double-arrowed line indicates larger effect size of the positive allele than the negative allele. Red double-arrowed line indicates larger effect size of the negative allele than the positive allele.

The 6528 additive effects were distributed over all chromosomes (Figure 1a, Table S1). SNP *rs108997231* in the *SLX4IP* gene of Chr13 had the most significant additive effect with log_10_(1/p) = 30.49, followed by SNPs on chromosomes 1, 17, 20, and 26 (Figure 1a, Table 1). In general, the difference between the statistical significance of any two additive effects ranked next to each other were relatively small. Therefore, the results of additive effects indicated genome-wide contributions of additive effects to DPR in the crossbred dairy cows in this study. Among the top 1000 significant SNP effects, negative allelic effects on average had larger sizes (average −0.957) than positive allelic effects (average 0.629), but the positive alleles had higher frequencies (average 0.60) than the frequencies of the negative alleles (average 0.40) (Table S1). SNP *rs42052893* in the *KDSR* gene of Chr24 had the largest positive allelic effect (2.62) with # 663 ranking in statistical significance, whereas SNP *rs110314053* upstream of *LOC107132214* of Chr01 had the largest negative allelic effect (–2.17) with #562 ranking in statistical significance (Table S1, Figure 1b). Four examples of the distribution of allelic effects of the top 1000 additive effects on different chromosomes are shown in Figure 1c–f. Compared to the Holstein DPR studies, this crossbred study had a larger number of additive effects but lacked chromosome regions that were much more significant than other chromosome regions. The 6528 additive effects in this crossbred study with 40,203 cows were more than six times as many as the 1126 additive effects from the 2019 Holstein study using 245,214 cows [15] that were more than nine times as many as the 40,203 cows in this crossbred study, and were only 14% fewer than the 7567 additive effects from the Holstein study using 1,194,736 cows [16] that were 29 times as many as the 40,203 cows in this crossbred study. Based on the comparison between the crossbred and the 2019 Holstein studies, the number of significant additive effects in the crossbred study was at least six times as many as in Holsteins, indicating that additive effects were a contributing factor to DPR heterosis in crossbred dairy cows.

### 2.2. Dominance Effects

**Figure 2:**
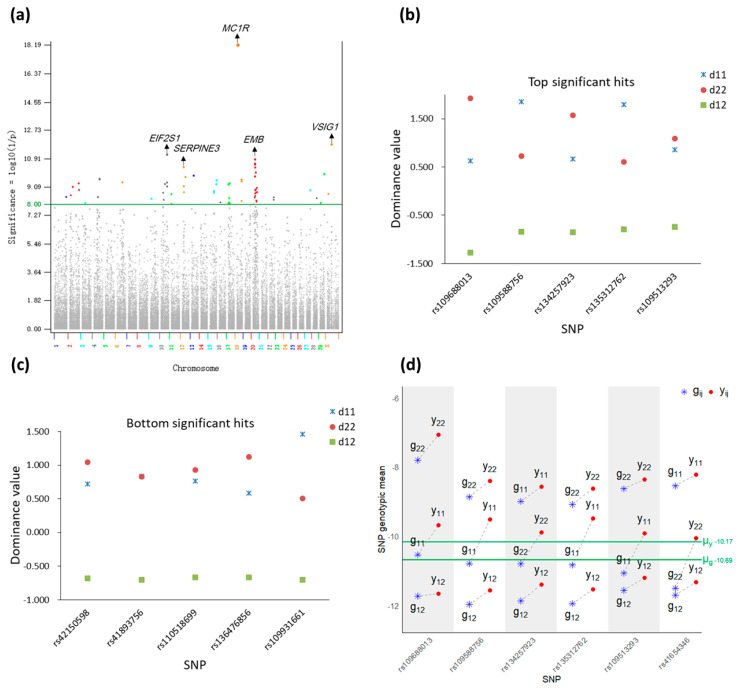
Dominance effects and the homozygous advantage and heterozygous disadvantage of significant dominance effects of DPR. (**a**) Manhattan plot of genome-wide dominance effects, showing that the *MC1R* gene had a far more significant dominance effect than in other regions; (**b**) dominance values of top-ranked significant dominance effects; (**c**) dominance values of bottom-ranked significant dominance effects; (**d**) examples of SNP genotypic mean of phenotypic values. d_ij_ is the dominance value of the ij SNP genotype, ij = 1,2. y_ij_ is the SNP genotypic mean of the original phenotypic values without removing pedigree additive (breeding) values. g_ij_ is the SNP genotypic mean of the corrected phenotypic values after removing pedigree additive (breeding) values. µ_y_ is the population mean of the original phenotypic values without removing pedigree additive (breeding) values. µ_g_ is the population mean of the corrected phenotypic values after removing pedigree additive (breeding) values.

The 65 dominance effects were distributed over 19 autosomes and the X chromosomes with Chr20 having the largest number (17) of dominance effects (Table S2, Figure 2a). SNP *rs109688013* in the *MC1R* gene of Chr18 had the most significant dominance effect with log_10_(1/p) = 18.19, the most positive dominance value (d_22_ = 1.92) and the most negative dominance value (d_12_ = –1.28), and the largest negative dominance effect, δ = d_12_ − 0.5 (d_11_ + d_22_) = –2.55. Other SNPs with significant dominance effects following *rs109688013* included SNPs on chromosomes X, 10, 20, and 12 (Figure 2a, Table 2). The number of dominance effects (65) is much smaller than the number of additive effects (6528) in this study but is far more than the two significant dominance effects from the 2019 Holstein study using 245,214 cows [15].

A striking feature of the dominance effects was the consistent pattern of homozygous advantage and heterozygous disadvantage according to the three dominance values (d_ij_) of each significant dominance effect (δ), showing that the homozygous genotypes all had positive dominance values, one of the two homozygous genotypes of each SNP had the highest dominance value, and the heterozygous SNP genotypes of all 65 SNPs had the lowest dominance values that were all negative (Figure 2b,c; Table S2). Since each dominance value is a deviation of the genotypic value from the population mean of all three genotypic values and the additive value of the SNP genotype, the pattern of each dominance value could be affected by the estimate of the additive value of the genotype. Therefore, the phenomenon of homozygous advantage and heterozygous disadvantage detected by dominance values was further validated by the genotypic means of two types of phenotypic values: the original phenotypic values without removing pedigree additive (breeding) values (y_ij_) and the corrected phenotypic values after removing pedigree additive (breeding) values (g_ij_). The results of the original phenotypic values confirmed the phenomenon of homozygous advantage and heterozygous disadvantage for all 65 dominance effects, i.e., y_11_ or y_22_ had the highest genotypic mean whereas y_12_ had the lowest genotypic mean. The results of the corrected phenotypic values had only four SNPs that did not have the lowest genotypic means for the heterozygous genotypes (g_12_) but one of the homozygous genotypes of every SNP was still the most positive genotype for all 65 dominance effects. Therefore, the dominance and original phenotypic values confirmed that all dominance effects had homozygous advantage and heterozygous disadvantage whereas the corrected phenotypic values confirmed homozygous advantage for all 65 dominance effects and heterozygous disadvantage for 61 of the 65 dominance effects (Figure 2d, Table S2).

### 2.3. Additive × Additive (A × A) Epistasis Effects

**Figure 3:**
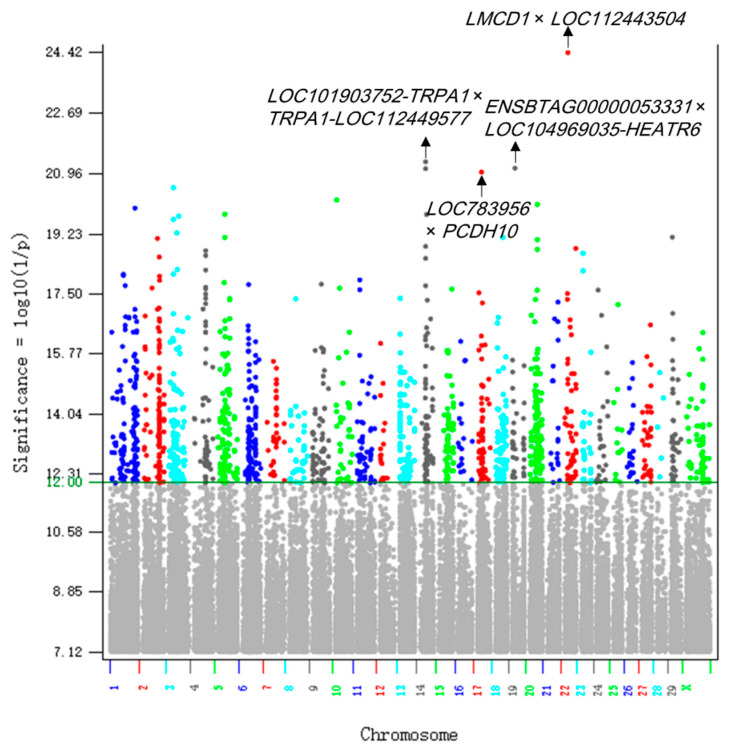
Manhattan plot of A × A epistasis effects. The significant A × A epistasis effects were distributed on all chromosomes but lacked chromosome regions with A × A effects far more significant than in other regions.

The 1658 significant A × A effects detected in the 40,203 crossbred cows were far more than just one significant A × A effect detected in the 2021 Holstein study using 245,214 cows [17]. Of the 1658 A × A effects, 1654 were intra-chromosome and four inter-chromosome A × A effects (Table S3). The 1658 A × A epistasis effects were distributed on all chromosomes but lacked chromosome regions with A × A effects far more significant than in other regions (Figure 3). The significance and distribution patterns of the A × A effects indicated genome-wide contributions of A × A effects to DPR heterosis, like the contributions of the additive effects. Although all chromosomes had intra-chromosome A × A epistasis effects, different chromosomes had large variations in the number of intra-chromosome A × A effects, e.g., Ch20 had 110 effects whereas Chr28 only had five effects. The distance between two interacting SNPs on the same chromosome was in the range of 6702 bp to 2.68 Mb, and many interacting SNP pairs were within the same genes. The A × A epistasis effects had an interesting pattern: the most positive and the most negative allelic combinations always involved one allele of one SNP and two alleles at the other interacting SNP (Table S3). The top 20 A × A effects are shown in Table 3.

**Figure 4:**
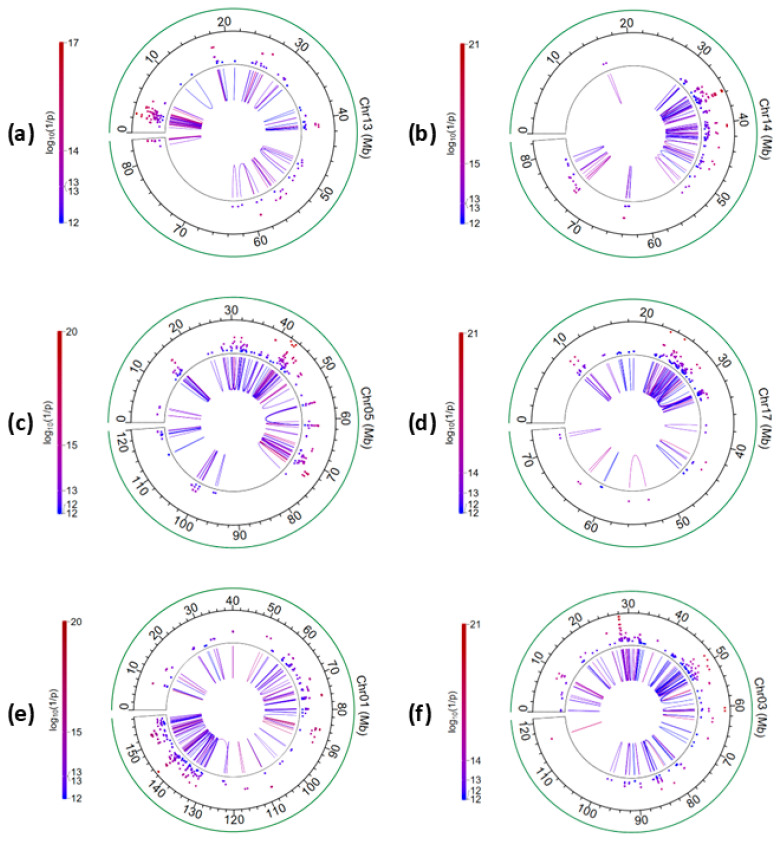
Circular plot of intra-chromosome A × A epistasis effects for six selected chromosomes. (**a**) The 1.08–3.71 Mb region of Chr13 had 31 pairs of A × A epistasis effects with the *PLCB1* and *SLX4IP* genes at the two ends of this region; (**b**) the 34–74 Mb region of Chr05 had a large cluster of A × A effects; (**c**) the 31–51 Mb region of Chr14 had a large cluster of A × A effects; (**d**) the 22–32 Mb region of Chr17 had a large cluster of A × A effects; (**e**) Chr01 had A × A effects covering over half of the chromosome; (**f**) Chr03 had A × A effects covering over half of the chromosome.

Within each chromosome, some chromosome regions had large concentrations of intra-chromosome A × A epistasis effects and six examples of such large concentrations are shown in Figure 4. The 1.08–3.71 Mb region of Chr13 had 31 pairs of A × A epistasis effects mostly involving six genes, *PLCB1, PLCB4, PAK5, ANKEF1, SNAP25*, and *SLX4IP* (Table S3, Figure 4a). Through interaction with neighboring genes, the 31 pairs of A × A epistasis effects formed an interacting chromosome region about 2.63 Mb in size. It was interesting to note that the two genes at the two ends of this region, *PLCB1* at the upstream end and *SLX4IP* at the downstream end, were reported to be associated with fertility, with *PLCB1* affecting embryonic implantation failure in mice and *SLX4IP* affecting embryogenesis in zebrafish [18]. It was also interesting to note that *SLX4IP* had the most significant additive effect in this study. The 31–51 Mb region of Chr14 (Figure 4b), the 24–74Mb region of Chr05 (Figure 4c), and the 22–32 Mb region of Chr17 (Figure 4d) each had a large cluster of A × A epistasis effects; Chr01 (Figure 4e) and Chr03 (Figure 4f) each had A × A epistasis effects covering over half of the chromosome.

Only four significant inter-chromosome A × A effects were detected between Chr02 and Chr12, Chr08 and Chr19, Chr07 and Chr18, and Chr20 and Chr26 (Table S3). The A × A values of the four inter-chromosome A × A effects all had the same pattern as that of the intra-chromosome: the most positive and negative A × A values were due to interactions between one allele of one SNP and two alleles of the other interacting SNP.

### 2.4. Additive × Dominance (A × D and D × A) Epistasis Effects

The GWAS analysis detected two intra-chromosome A × D effects on Chr20 and one inter-chromosome and D × A effect between Chr04 and Chr19 (Table S4). The *HECW1* gene with a D × A effect also had two other SNPs with an intra-chromosome A × A effect. Although these results showed the existence of A × D and D × A effects in the crossbred cows, A × D and D × A effects were unlikely to be an important mechanism underlying DPR heterosis in crossbred cows due to the small number of the A × D and D × A effects.

### 2.5. Dominance × Dominance (D × D) Epistasis Effects

The GWAS analysis detected 18 intra-chromosome D × D effects on nine chromosomes with Chr20 having the largest number (six) of D × D effects (Table 4). The chromosome distance between two interaction loci was in the range of 3.61 Kb to 1.77 Mb, and only one pair of interacting loci were within the same gene, *MAGI1* of Chr22. All 18 D × D effects had a heterozygous disadvantage where any two-locus genotypes with a heterozygous genotype at one locus and a homozygous genotype at the other locus had a negative D × D value. The doubly heterozygous genotype and 68 of the 72 two-locus homozygous genotypes had positive D × D values (Table S5). These D × D results added evidence to the phenomenon of heterozygous disadvantage and homozygous advantage observed for single-locus dominance effects. The doubly heterozygous genotype (*AaBb*) in fact had a positive value and was an interesting exception of the heterozygous disadvantage. Of the 18 doubly heterozygous genotypes, seven had DD3 values (3rd most positive values), eight had DD4 values, and one had a DD5 value, noting that all 18 DD3 and 18 DD4 values were positive, and DD5 had fourteen positive values and four negative values (Table S5). The positive D × D values of *AaBb* underwent negative switching twice: the heterozygous genotype at one locus turned a positive value into a negative value and the heterozygous genotype at the other locus turned the negative value back to positive, but the actual reason for the positivity of *AaBb* is yet to be understood.

### 2.6. Genetic Mechanism of DPR Heterosis in Dairy Cattle

The GWAS results indicated that the genetic mechanism of DPR heterosis in crossbred dairy cows included a larger collection of favorable alleles than in Holsteins, and allelic and genotypic interactions. These genetic mechanisms can be summarized as the following: complementary additive effects from different breeds, allelic interactions between different breeds, homozygous advantage due to interaction between the same alleles from different breeds and heterozygous disadvantage, and genotype × genotype interactions.

#### 2.6.1. Complementary Additive Effects from Different Breeds and New Additive Effects Due to Cross Breeding

**Figure 5:**
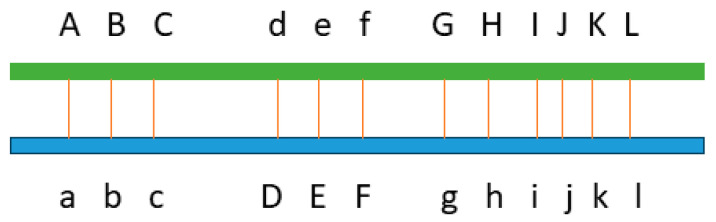
Complementary additive effects between two breeds. Capital letter represents favorable allele, and lower-case letter represents unfavorable or neutral allele. Green represents one breed, and blue represents the other breed. The green breed has nine favorable alleles, the blue breed has three favorable alleles, and the crossbred between the two breeds has 12 favorable alleles.

This crossbred study with 40,203 cows had more than six times as many additive effects as the Holstein study on additive and dominance effects using 245,214 cows [15], as described earlier. This result supports the hypothesis that the large number of additive effects were due to complementary additive effects between different breeds, where a chromosome region lacked favorable alleles in one breed, but another breed had favorable alleles in the same region. For the hypothetical example of Figure 5, the chromosome region from the breed represented in green has nine favorable alleles whereas the breed represented in blue has only three favorable alleles in the same region, but the crossbred from these two breeds has 12 favorable alleles, more than those in either breed. The distribution of the additive effects over the entire genome indicated that chromosome regions with complementary additive effects from different breeds widely existed on the cattle genome. Given that Holsteins and Jerseys had the largest genetic contributions (combined 97.11% breed contributions, see Section 3) to the crossbred cows, the additive results indicated the broad existence of complementary additive effects between Holsteins and Jerseys. However, the summation of additive effects in both Holsteins and Jerseys will unlikely reach the number of additive effects observed in this study, noting that the number of additive effects for the DPR in Jerseys was unknown. Therefore, cross breeding likely activated new additive effects that were either undetectable or non-existent in the pure breeds.

#### 2.6.2. Two-Locus Allelic Interactions Between Loci and Between Breeds

This crossbred study with 40,203 cows detected 1658 significant additive A × A effects, compared to only one significant A × A effect from the Holstein study on epistasis effects using 245,214 cows [17], and the only significant Holstein A × A effect was not detected in the crossbred cows. As the genetic effect type with the second largest number of significant effects, A × A effects were the second most common effects in the crossbred cows after additive effects. Two-locus allelic interactions are the genetic interpretation of pairwise A × A effects and such allelic interactions in this study were allelic interactions between breeds.

**Figure 6:**
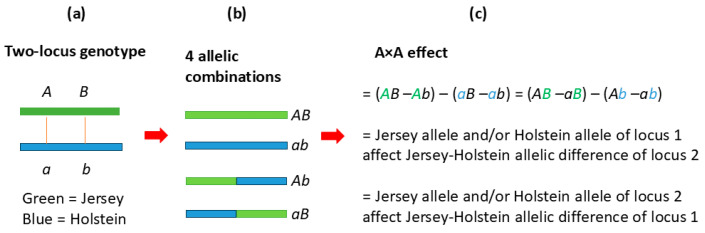
A × A effects in crossbred cows are allelic interaction effects between two loci.

A significant A × A effect requires at least one pair of alleles, one allele from each locus interacting with each other as described in Figure 6. For two biallelic loci with each locus having one allele from Jersey and one allele from Holstein (Figure 6a), four allelic combinations are possible, *AB* from Jersey, *ab* from Holstein, and the *Ab* and *aB* allelic combinations each have one allele from Jersey and one allele from Holstein (Figure 6b). The A × A effect of the two loci is a contrast of the mean genotypic values of the four allelic combinations in the population, and allelic interactions must be present for at least one of the four allelic combinations for the A × A effect to be statistically significant (Figure 6c). Although the method in this study does not identify which alleles are interacting with each other, the interacting alleles could be deduced. Since none of the A × A effects in crossbred cows was detected in Holstein cows, the *ab* allelic combination from Holstein could be assumed to have no interaction between the two alleles. Since Jersey epistasis study was unavailable and Jersey also had lower DPR levels than in crossbred cows, it should be reasonable to assume most of the A × A effects in the crossbred cows were insignificant in Jersey cows. Under this assumption, the *AB* allelic combination from Jersey also had no interaction between the two alleles, leaving only *Ab* and *aB* allelic combinations to be the candidates with allelic interactions responsible for the significant A × A effect, noting that each of these two allelic combinations has one allele from Jersy for one locus and one allele from Holstein for the other locus. Therefore, the mostly likely reason for the significant A × A effects in the crossbred cows was allelic interactions between loci and between breeds.

#### 2.6.3. Within-Locus Allelic Interaction Between Different Breeds Underlying Homozygous Advantage and Heterozygous Disadvantage

**Figure 7:**
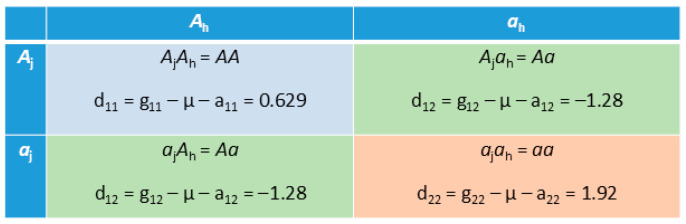
Example of dominance values (deviations) of crossbred cows from Jersey–Holstein cross. Jersey alleles are *A*_j_ and *a*_j_ and Holstein alleles are *A*_h_ and *a*_h_. d_ij_ = dominance value (deviation), g_ij_ = genotypic value, a_ij_ = additive value, i, j = 1, 2, µ = population mean of the three genotypic values.

Within-locus allelic interactions of a biallelic SNP locus are reflected in the dominance values of the three genotypes of the locus including the two homozygous genotypes and the heterozygous genotype. Assuming a Jersey–Holstein cross, each SNP genotype of a crossbred cow consists of one Jersey allele and one Holstein allele (Figure 7). The dominance value (d_ij_) of a genotype is the deviation of the genotypic value (g_ij_) from the additive value (a_ij_) of the genotype and the population average of all three genotypic values (µ), where the additive value of the genotype is the sum of the two allelic effects. A large dominance value of a genotype, positive or negative, indicates the presence of allelic interactions within the genotype. The example of Figure 7 is the dominance values of *rs109688013* in the *MC1R* gene of Chr18 with the most significant dominance effect (Table 2 and Table S2). The two homozygous genotypes (*A*_j_*A*_h_ or *a*_j_*a*_h_) had positive dominance values (d_22_ = 1.92 and d_11_ = 0.629) and the heterozygous genotype (*a*_j_*A*_h_ or *A*_j_*a*_h_, not distinguishable in this study) had a negative dominance value (d_12_ = –1.28). Since the two alleles of every genotype originated from different breeds, the within-locus allelic interactions were allelic interactions between different breeds. These dominance values indicated that every genotype had within-locus allelic interaction between breeds that was responsible for the homozygous advantage and heterozygous disadvantage. All the advantages and disadvantages revealed by the dominance values were further confirmed by the original and corrected phenotypic values, as shown in Figure 2d. The interaction between two same alleles from different breeds within a homozygous genotype (*A*_j_*A*_h_ or *a*_j_*a*_h_) was interesting because the concept of dominance is typically associated with the heterozygous genotype. For this reason, the terminology ‘within-locus interaction’ is a more general description than ‘dominance’ for interpreting the dominance values, particularly those of the homozygous genotypes. The quantitative genetics approach of dominance values in this study led to the finding of allelic interactions between breeds within both homozygous and heterozygous genotypes but had no indication as to why two same alleles from different breeds interacted with each other or why homozygous genotypes had advantages and heterozygous genotypes had disadvantages. Compared to the additive and A × A effects, the number of significant dominance effects is relatively small and should have limited contributions to the DPR heterosis.

#### 2.6.4. Genotype × Genotype Interactions Enabled by Allelic Interactions Between Breeds

**Figure 8:**
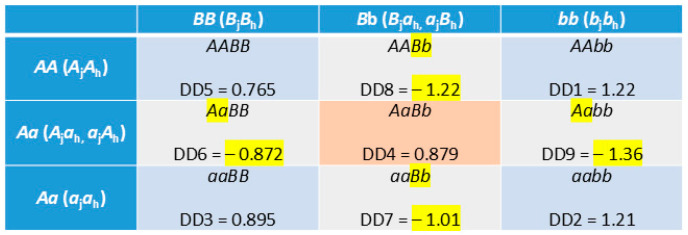
Example of genotype × genotype interactions for different D × D values of nine two-locus SNP genotypes. The two alleles of each SNP assumed to originate from Jersey are indicated by subscript j and Holstein are indicated by subscript h. Every two-locus genotype with a heterozygous genotype at either SNP (highlighted in yellow) had a negative D × D value (highlighted in yellow), whereas the doubly heterozygous genotype (*AaBb*) and all homozygous genotypes had positive D × D values. DD1–DD9 are the nine D × D values of the most significant D × D effect from Table S5.

Genotype × genotype (G × G) interactions are the reasons for the 18 significant D × D effects which also had homozygous advantage and heterozygous advantage. Figure 8 is the example of the most significant D × D effect between *rs42670220* and *rs41942366* of Chr20 showing G × G interactions reflected in the nine D × D values of DD1-DD9 ranked from the most positive to the most negative. Any two-locus genotype with a heterozygous genotype at one locus and a homozygous genotype at the other locus was negative (highlighted in yellow) and all homozygous genotypes were positive. The D × D values clearly demonstrated the presence of G × G interactions in crossbred cows. The *AAbb* genotype had the most positive D × D value of DD1 = 1.22, but *AA* and *bb* individually were also associated with very negative D × D values: the *Aabb* genotype had the most negative D × D value of DD9 = –1.36 and the *AABb* genotype had the second most negative D × D value of DD8 = –1.22. Since parents do not transmit any genotype to the next generations and each breed transmits only one allele per locus to the offspring, the G × G interactions were not direct interactions between breeds but were enabled by the allelic interactions between the parental breeds. Moreover, no G × G interaction was detected in the 2021 Holstein study using 245,214 cows [17]. Therefore, the G × G interaction in crossbred cows enabled by the allelic interactions between the parental breeds was a genetic mechanism underlying DPR heterosis in crossbred cows.

### 2.7. Candidate Genes with Fertility and Reproductive Functions

The significant genetic effects detected in this study were associated with many candidate genes known to have fertility and reproductive functions. We searched for such genes among the top 20 additive effects, the top 20 dominance effects, the top 20 intra-chromosome A × A effects, the three inter-chromosome A × A effects, the three A × D and D × A effects’ top 20 dominance effects, and the 18 D × D effects. In addition, we noted that some genes with highly significant DPR effects in Holstein cows [15,16] were also significant in the crossbred cows, although the effects of those genes in crossbred cows were not highly ranked. Together, 82 candidate genes known to affect many aspects of fertility and reproduction were identified (Appendix A), indicating that fertility and reproductive genes likely were broadly involved in DPR heterosis in crossbred dairy cows. Important fertility and reproductive functions of the candidate genes included embryo development (*KALRN*, *DAZL*, *IFNG*, *SERPINE3*, *BMP7*, *PCDH10*, *FOXC2*, *SIPA1L3*, *NDUFS4*, *GHR*, *DROSHA*, *EOMES*, *KDSR*, and *RARB*), embryonic implantation failure (*PCLB1*), embryogenesis (*SLX4IP*), maintenance of pregnancy (*IGFBP7* and *IGFBP3*), placentation and pregnancy maintenance (*EIF2S1*), uterine contraction and pregnancy maintenance (*OXTR*), progesterone synthesis (*FGF12*), early pregnancy (*SLC4A4*), delayed puberty (*DLG2*), oogenesis (*HECW1* and *LARP1B*), oocyte maturation and female fertility (*CNOT2*), sex hormone production (*DIAPH1*), gonadotropin-releasing hormone (*HOMER1*), growth hormone (*GHR*), and thyroid hormone (*THRB*). Some genes with highly significant effects on the Holstein DPR were also significant but not ranked high in the crossbred cows, including *SLC4A4* and *GC* of Chr06, *KALRN* of Chr01, and *PEPD*, *CHST8,* and *SIPA1L3* of Chr18. The *KALRN*, *PEPD*, and *CHST8* genes had extremely negative recessive alleles for the DPR in Holstein cows [16] but those extremely negative effects were not observed in the crossbred cows.

## 3. Materials and Methods

### 3.1. Dairy Crossbred Population and SNP Data

The dairy crossbred population in this study had 40,203 cows with first lactation phenotypic observations on DPR resulting from crosses among five dairy breeds: Ayrshire, Brown Swiss, Guernsey, Holstein, and Jersey. Based on breed base representation (BBR), a measure for identifying breed origin, a cow or bull is considered a purebred animal if the animal’s BBR values is 94–100% [19]. The breed contributions to the 40,203 crossbred cows measured by average BBR per breed as the percentage of all BBR values of the five breeds were 51.80% from Jersey, 45.31% Holstein, 1.60% Ayrshire, 0.99% Brow Swiss, and 0.30% from Guernsey. Therefore, the crossbreeds were primarily due to the Jersey × Holstein cross given that Jersey and Holstein had a combined 97.11% breed contribution to the crossbred cows. Ignoring the ‘0’ BBR values that every breed had, the range of the BBR values was 2.5–89.5% for Jersey, Holstein, Ayrshire, and Brown Swiss; and 2.5–89.2% for Guernsey, indicating varying degrees of purebred genetic contributions among different crossbred cows that should provide necessary contrasts between favorable and unfavorable genetic factors underlying DPR in the crossbred cows. The phenotypic values used in the GWAS analysis were the phenotypic residuals after removing fixed non-genetic effects available from the December 2023 U.S. Holstein genomic evaluation by the Council on Dairy Cattle Breeding (CDCB). Among Holstein, Jersey, and crossbred cows, crossbreds had the highest average DPR values (54.97), followed by Jersey (53.43) and Holstein (49.06) (Table 5). Since 1% DPR is equivalent to four fewer days open, crossbred cows on average had about 24 fewer days open than Holstein cows and six fewer days open than Jersey cows, showing that crossbred cows had DPR heterosis over Holstein and Jersey cows. The SNP genotypes were from 32 SNP chips with various densities and were imputed into 78,964 SNPs via the FindHap algorithm [20] as a routine procedure for genomic evaluation by CDCB [21]. The SNP genotyping quality control by CDCB had checks and requirements at the individual and SNP levels, including call rate, parent–progeny conflicts, sex verification using X-specific SNPs, and Hardy-Weinberg equilibrium [22,23]. In addition, we applied minor allele frequency (MAF) of 5% for SNP filtering in this study. With the requirement of 5% MAF, the number of SNPs for the GWAS analysis was 75,133. The threshold p-value for declaring significant effects for the Bonferroni correction with 0.05 genome-wide false positives was 0.05/[(2)(75,133)] = 10^−8^ or log_10_(1/p) = 8 for additive and dominance effects and was 0.05/[(4)(75,133)(75,132)/2] = 10^−12^ or log_10_(1/p) = 12 for pairwise epistasis effects. The SNP and gene positions were those from the ARS-UCD1.3 cattle genome assembly [24]. Genes containing or in proximity to highly significant SNP effects were identified as candidate genes for DPR in the crossbred dairy cows.

### 3.2. GWAS Analysis

The GWAS analysis used an approximate generalized least squares (AGLS) method. The AGLS method combines the least squares (LS) tests implemented by EPISNP1mpi [25,26] with the phenotypic correction using the estimated breeding values from routine genetic evaluation using the entire U.S. Holstein population. For each SNP pair, the statistical model was

**y** = µ**1 **+** X**_g_**g **+** Za **+** e = Xb **+** Za **+** e** (1)

where **y** = column vector of phenotypic deviation after removing fixed nongenetic effects such as heard–year–season (termed as ‘yield deviation’ for any trait) using a standard procedure for the CDCB genetic and genomic evaluation; µ = common mean; **1** = column vector of 1’s; **g** = column vector of genotypic values of the nine genotypes of the SNP pair; **X**_g_ = model matrix of **g**; b=(μ,g′)′; **X** = (**1**, **X**_g_); **a** = column vector of pedigree additive values; **Z** = model matrix of **a**; and **e** = column vector of random residuals. The first and second moments of Equation (1) are E(**y**) = **Xb** and $var\left(y\right)=V=ZGZ^{\prime }+R=\sigma_{a}^{2}ZAZ^{\prime }+\sigma_{e}^{2}I$, where $\sigma_{a}^{2}$ = additive variance, **A** = additive relationship matrix, $\sigma_{e}^{2}\;$= residual variance, and **I** = identity matrix. The problem of estimating the **b** vector that includes SNP genotypic values in Equation (1) is the requirement of inverting the **V** if the generalized least squares (GLS) method is used or solving the mixed model equations (MMEs) [27]. Either the GLS or MME method for each of the genome-wide SNPs is computationally challenging. To avoid these computing difficulties, the GWAS used the AGLS method that replaces the pedigree additive values (**a**) with the best linear unbiased prediction based on pedigree relationships [15] that were readily available from CDCB’s routine genetic and genomic evaluations. The AGLS method is based on the following results:

(2) $$\hat{b}={\left(X\prime V^{-1}X\right)}^{-}X\prime V^{-1}y$$

(3) $$\begin{array}{ll} \hat{b} & ={\left(X^{\prime }R^{-1}X\right)}^{-}\left(X^{\prime }R^{-1}y-X^{\prime }R^{-1}Z\hat{a}\right)={\left(X^{\prime }X\right)}^{-}X^{\prime }(y-Z\hat{a})\\ & ={\left(X^{\prime }X\right)}^{-}X\prime y^{*} \end{array}$$

where $y^{*}=y-Z\hat{a}$ and $\hat{a}$ is the best linear unbiased prediction (BLUP) of **a**. Equation (2) is the GLS solution and Equation (3) is the MME solution of **b**. These two equations yield identical results and $\hat{b}$ from either equation is termed the best linear unbiased estimator (BLUE) [27]. If $\hat{a}$ is known, the LS version of BLUE given by Equation (3) is computationally efficient relative to the GLS of Equation (2) that requires the **V** inverse or the joint MME solutions of $\hat{b}$ and $\hat{a}$. The AGLS method uses two approximations. The first approximation is to use $\tilde{a}$ from routine genetic evaluation as an approximation of $\hat{a}$ in Equation (3):

(4) $$\hat{b}={\left(X^{\prime }X\right)}^{-}X\prime \left(y-Z\tilde{a}\right)={\left(X^{\prime }X\right)}^{-}X\prime y^{*}$$

where $y^{*}=y-Z\tilde{a}$ and $\tilde{a}$ is the column vector of 2(PTA) with PTA being the predicted transmitting ability from the routine genetic evaluation. Equation (4) achieves the benefit of sample stratification correction from mixed models using pedigree relationships without the computing difficulty of inverting **V** or the joint MME solutions of $\hat{b}$ and $\hat{a}$ for every SNP. The second approximation of the AGLS approach is the t-test using the LS rather than the GLS formula of the t-statistic to avoid using the **V** inverse in the GLS formula. The significance tests were used for the t-tests of the contrasts of the estimated SNP genotypic values of the nine genotypes of each SNP pair where locus 1 had *AA*, *Aa*, and *aa* genotypes and locus 2 had *BB, Bb*, and *bb* genotypes [25,28]. The t-statistic of the AGLS was calculated as

(5) $$t_{j}=\frac{\left|L_{j}\right|}{\sqrt{var(L_{j})}}=\frac{\left|s_{j}\hat{g}\right|}{v\sqrt{s_{j}{\left(X\prime X\right)}_{gg}^{-}s_{j}^{\prime }}},\;j=A,D,A\times A,A\times D,D\times A,D\times D$$

where L_j_ = effect contrast; $\sqrt{var(L_{j})}\;$= standard deviation of the effect contrast; $s_{j}$ = row vector of the contrast coefficients for the $j^{th}$ effect type; $\hat{g}$ = column vector of the AGLS estimates of the nine SNP genotypic values for two loci from Equation (4); A = additive; D = dominance; A × A = additive × additive; A × D = additive × dominance; D × A = dominance × additive; D × D = dominance × dominance; $v^{2}=(y-X\hat{b})\prime (y-X\hat{b})/(n-k)\;$= estimated residual variance; ${\left(X\prime X\right)}_{gg}^{-}$ = submatrix of ${\left(X^{\prime }X\right)}^{-}$ corresponding to $\hat{g}$; n = number of observations; and k = rank of **X**. The formula of $s_{a}$ defined above allows Hardy-Weinberg disequilibrium [28]. To understand the genetic effects, the genetic values for each effect contrast (L_j_) were calculated based on Kempthorne’s definitions [29,30] with an extension to allow for Hardy–Weinberg and linkage disequilibria [28] implemented by the EPISNP1mpi computing tool [25,26]:

(6) $$a_{i}={µ}_{i}-\mu ,\;\;\;\;i=A,\;a$$

(7) $$a_{k}={µ}_{k}-\mu ,\;\;\;\;k=B,\;b$$

(8) $$d_{ij}={µ}_{ij}-\mu -a_{i}-a_{j},\;\;\;\mathrm{ij}=\mathrm{AA},\;\mathrm{Aa},\;\mathrm{aa}$$

(9) $$d_{kl}={µ}_{kl}-\mu -a_{k}-a_{l},\;\;\;\mathrm{kl}=\mathrm{BB},\;\mathrm{Bb},\;\mathrm{bb}$$

(10) $$a_{ik}={µ}_{ik}-\mu -a_{i}-a_{k},\;\;\;\mathrm{ik}=\mathrm{AB},\;\mathrm{Ab},\;\mathrm{aB},\;\mathrm{ab}$$

(11) $${\left(ad\right)}_{ikl}={µ}_{ikl}-\mu \;-\;a_{i}-a_{k}-a_{l}-d_{kl}-{\left(aa\right)}_{ik}-{\left(aa\right)}_{il}\;i=A,\;a;\;\mathrm{kl}=\mathrm{BB},\;\mathrm{Bb},\;\mathrm{bb}$$

(12) $${\left(da\right)}_{ijk}={µ}_{ijk}\;-\;\mu \;-\;a_{i}-a_{j}-a_{k}-d_{ij}-{\left(aa\right)}_{ik}-{\left(aa\right)}_{jk},\;\mathrm{ij}=\mathrm{AA},\;\mathrm{Aa},\;\mathrm{aa};\;k=B,\;b$$

(13) $${\left(dd\right)}_{ijkl}=g_{ijkl}-\mu -a_{i}-a_{j}-\;a_{k}-a_{l}-d_{ij}-d_{kl}\;-\;{\left(aa\right)}_{ik}-{\left(aa\right)}_{il}-\;{\left(aa\right)}_{jk}-{\left(aa\right)}_{il}-{\left(ad\right)}_{ikl}-{\left(ad\right)}_{jkl}-{\left(da\right)}_{ijk}-{\left(da\right)}_{ijl},\;\mathrm{ij}=\mathrm{AA},\;\mathrm{Aa},\;\mathrm{aa};\;\mathrm{kl}=\mathrm{BB},\;\mathrm{Bb},\;\mathrm{bb}$$

where $a_{i}$ = allelic effect of allele i at locus 1 (i = *A*, *a*), $a_{k}$ = allelic effect of allele k at locus 2 (k = *B*, *b*), $d_{ij}$ = dominance value of genotype ij at locus 1, $d_{kl}$ = dominance value of genotype kl at locus 2, (*aa*)*_ik_* = additive × additive epistasis value of genotypes with alleles ik, ${\left(ad\right)}_{ikl}$ = additive × dominance epistasis value of genotypes with alleles ikl, ${\left(da\right)}_{ijk}$ = dominance × additive epistasis value of genotypes with alleles ijk, ${\left(dd\right)}_{ijkl}$ = dominance × dominance epistasis value of ijkl genotype, μ = the mean genotypic value in the population, ${µ}_{i}$ = the marginal mean of genotypic values for individuals with allele i (i = *A*, *a*), ${µ}_{k}$ = the marginal mean of genotypic values for individuals with allele *k* (k = *B*, *b*), ${µ}_{ij}$ = the marginal mean of genotypic values for individuals with genotype ij at locus 1 (ij = *AA*, *Aa*, and *aa*), ${µ}_{ik}$ = the marginal mean of genotypic values for individuals with genotype kl at locus 2 (kl = *BB*, *Bb*, and *bb*), ${µ}_{ik}$ = the marginal mean of genotypic values for individuals with allele i at locus 1 and allele k at locus 2 (i = *A*, *a*; k = *B*, *b*), ${µ}_{ikl}$ = the marginal mean of genotypic values for individuals with allele i at locus 1 and genotype kl at locus 2 *(i* = *A*, *a*; kl = *BB*, *Bb*, and *bb*), and ${µ}_{ijk}$ = the marginal mean of genotypic values for individuals with genotype ij at locus 1 and allele k at locus 2 (ij = *AA*, *Aa*, *aa*; k = *B*, *b*).

## 4. Conclusions

The GWAS using 40,203 crossbred dairy cows showed that genome-wide complementary additive effects between breeds and A × A epistasis effects due to between-breed and between-locus allelic interactions were the primary genetic mechanism of DPR heterosis. Interesting and complex genetic mechanisms of DPR heterosis were observed, including dominance effects due to between-breed and within-locus allelic interactions with homozygous advantage and heterozygous disadvantage and D × D effects enabled by between-breed and between-locus allelic interactions also with homozygous advantage and heterozygous disadvantage.

## Figures and Tables

**Table 1:** Top 20 additive effects of daughter pregnancy rate (DPR).

SNP	Chr	Position	Candidate Gene	α	al+	ae+	f_al+	al-	ae-	f_al-	log_10_(1/p)
*rs108997231*	13	3906114	*SLX4IP*	2.116	1	0.966	0.543	2	−1.150	0.457	30.49
*rs110789800*	1	76375060	*LOC614619*; *GMNC*	−2.005	2	0.655	0.673	1	−1.350	0.327	27.00
*rs3423093496*	26	29435606	*MIR2285K-4*(d)	−1.897	2	0.847	0.553	1	−1.050	0.447	26.69
*rs109778958*	17	29595964	*LARP1B*	1.928	1	0.698	0.638	2	−1.230	0.362	26.49
*rs41638404*	20	25229625	*ARL15*; *NDUFS4*	1.870	1	0.750	0.600	2	−1.120	0.400	26.30
*rs110560119*	26	22679137	*ARMH3*	1.835	1	0.735	0.599	2	−1.100	0.401	25.19
*rs43292973*	2	22416125	*CIR1*	1.870	1	0.790	0.577	2	−1.080	0.423	25.10
*rs111018678*	14	3308936	*TRAPPC9*	−1.913	2	0.553	0.711	1	−1.360	0.289	24.43
*rs109798047*	10	5830788	*LOC100138889*; *LOC781028*	−1.845	2	0.958	0.481	1	−0.887	0.519	24.41
*rs110511554*	10	36374183	*PPP1R14D*	1.856	1	0.991	0.466	2	−0.865	0.534	24.33
*rs110600324*	15	75950150	*PEX16*	1.869	1	1.070	0.427	2	−0.799	0.573	23.80
*rs43438147*	5	43898744	*FRS2*	−1.781	2	0.681	0.617	1	−1.100	0.383	23.65
*rs3423141818*	2	103153421	*ABCA12*	−1.819	2	0.845	0.535	1	−0.974	0.465	23.64
*rs3423165011*	3	118672880	*LOC101907483*; *LOC112446044*	1.811	1	0.651	0.640	2	−1.160	0.360	23.63
*rs43708865*	9	45754622	*LOC785087*; *LOC112448181*	−1.969	2	0.589	0.701	1	−1.380	0.299	23.59
*rs29019531*	1	97643741	*LOC112448275*; *LOC107132196*	−1.718	2	0.809	0.529	1	−0.909	0.471	23.45
*rs110080847*	24	21456850	*GALNT1*; *INO80C*	−1.944	2	0.674	0.653	1	−1.270	0.347	23.40
*rs43464313*	6	48853315	*LOC100298058*; *LOC112447186*	1.790	1	0.710	0.603	2	−1.080	0.397	23.37
*rs3423092932*	14	1854457	*ADGRB1*	1.757	1	0.597	0.660	2	−1.160	0.340	23.09
*rs42438948*	17	7548169	*LRBA*	−1.883	2	0.633	0.663	1	−1.250	0.337	22.87

‘d’ indicates that the SNP is downstream of the gene. ‘α’ is the additive effect of the SNP as the difference between allelic effects of ‘allele 1’ and ‘allele 2’. ‘ae+’ is the allelic effect of the positive allele. ‘ae-’ is the allelic effect of the negative allele. ‘f_al+’ is the frequency of the positive allele. ‘f_al-’ is the frequency of the negative allele.

**Table 2:** Top 20 dominance effects of daughter pregnancy rate (DPR).

SNP	Chr	Position	Candidate Gene	δ	d_12	f_12	d_11	f_11	d_22	f_22	log_10_(1/p)
*rs109688013*	18	14705671	*MC1R*	−2.555	−1.280	0.421	0.629	0.445	1.920	0.134	18.19
*rs109588756*	X	56705838	*VSIG1*	−2.136	−0.846	0.529	1.850	0.092	0.730	0.379	11.83
*rs134257923*	10	79265033	*EIF2S1*	−1.973	−0.854	0.511	0.667	0.368	1.570	0.121	11.17
*rs135634336*	20	27116615	*-*	−1.826	−0.784	0.563	0.953	0.292	1.130	0.145	10.87
*rs109658603*	20	26870070	*-*	−1.873	−0.775	0.560	1.340	0.121	0.855	0.319	10.60
*rs29018884*	20	27087053	*-*	−1.848	−0.767	0.559	0.841	0.320	1.320	0.121	10.54
*rs110147881*	12	20815304	*SERPINE3*	−1.751	−0.783	0.553	0.973	0.183	0.962	0.264	10.37
*rs135312762*	20	28614378	*EMB*	−1.988	−0.788	0.511	1.790	0.088	0.610	0.402	10.35
*rs111004899*	20	35745330	*LOC104975274*; *LOC101906686*	−1.742	−0.750	0.567	1.020	0.151	0.964	0.282	10.03
*rs109513293*	29	47636529	*SHANK2*	−1.714	−0.740	0.570	0.857	0.201	1.090	0.230	9.92
*rs41608172*	13	27232535	*TRNAE-UUC_63* (d)	−1.694	−0.752	0.554	0.971	0.168	0.912	0.278	9.83
*rs109628824*	20	27104916	*-*	−1.791	−0.745	0.559	0.821	0.320	1.270	0.121	9.78
*rs109680456*	12	42526721	*LOC107132995* (d)	−1.693	−0.713	0.571	0.660	0.235	1.300	0.193	9.74
*rs41653703*	4	76449707	*RAMP3*	−1.677	−0.729	0.567	0.924	0.165	0.972	0.268	9.63
*rs109588503*	4	76953836	*NUDCD3*	−1.675	−0.727	0.568	0.922	0.165	0.973	0.268	9.59
*rs133706373*	18	56085584	*PRMT1*	−1.723	−0.761	0.543	0.803	0.308	1.120	0.148	9.58
*rs41632642*	15	80951917	*SELENOH*	−1.651	−0.754	0.544	0.950	0.245	0.844	0.211	9.53
*rs41889435*	18	56040467	*PRR12*; *RRAS*	−1.659	−0.747	0.549	0.905	0.261	0.918	0.189	9.46
*rs41654346*	6	72581115	*IGFBP7*; *TRNAK-UUU_12*	−1.681	−0.754	0.536	0.773	0.314	1.080	0.150	9.40
*rs109489228*	10	78362097	*LOC112448513*	−1.930	−0.786	0.512	1.670	0.097	0.617	0.391	9.39

‘d’ indicates that the SNP is downstream of the gene. ‘δ’ is the dominance effect of the SNP as the difference between the heterozygous dominance value and the average of the two homozygous dominance values. ‘d_ij’ is the dominance value (deviation) of the ij SNP genotype. ‘f_ij’ is the frequency of the ij genotype, i = 1, 2 and j = 1, 2.

**Table 3:** Top 20 intra-chromosome A × A effects.

SNP-1	Chr	Position-1	Candidate Gene-1	SNP-2	Position-2	Candidate Gene-2	αα	log10(1/p)
*rs136164685*	22	17954298	*LMCD1*	*rs109133545*	17997196	*LOC112443504*	2.880	24.42
*rs3423093320*	14	35595531	*LOC101903752*; *TRPA1*	*rs3423093613*	35751572	*TRPA1*; *LOC112449577*	3.290	21.27
*rs41579785*	19	14094861	*ENSBTAG00000053331*	*rs3423464242*	14110279	*LOC104969035*; *HEATR6*	3.490	21.09
*rs3423363450*	14	35558190	*LOC101903752*; *TRPA1*	*rs3423093613*	35751572	*TRPA1*; *LOC112449577*	3.290	21.07
*rs133491240*	17	23169353	*LOC783956* (u)	*rs110348666*	25067804	*PCDH10*	2.600	20.97
*rs111002920*	3	27938935	*LOC782058*; *NGF*	*rs110020978*	28078792	*NGF*; *LOC112445967*	3.410	20.52
*rs41656697*	10	10474235	*HOMER1*	*rs29019953*	10520078	*HOMER1*	−2.350	20.16
*rs42982833*	20	45269875	*LOC112443070* (u)	*rs137619389*	45663409	*LOC112443070*; *LOC112442955*	3.330	20.04
*rs109874240*	1	142636491	*TFF1*; *TMPRSS3*	*rs42141252*	142787751	*SLC37A1*	−2.810	19.93
*rs109520109*	5	42304422	*CPNE8*	*rs43436966*	43506809	*LOC104972423*; *MYRFL*	−2.700	19.76
*rs134052004*	14	40390594	*PEX2*; *LOC101904449*	*rs41625934*	40600797	*LOC101904449*; *LOC782385*	−3.830	19.75
*rs42445061*	3	60310725	*TTLL7(d)*	*rs134212595*	60964021	*-*	2.470	19.70
*rs111002920*	3	27938935	*LOC782058*; *NGF*	*rs109896720*	28047336	*NGF*; *LOC112445967*	3.430	19.61
*rs109621977*	3	48432235	*ALG14*	*rs109849660*	49515221	*ABCA4*	−2.390	19.21
*rs109619154*	29	8688612	*LOC101905908*; *PRSS23*	*rs110088807*	9023536	*ME3*	2.920	19.09
*rs135072252*	18	40169055	*HYDIN*	*rs110785717*	40219814	*HYDIN*; *LOC112442400*	2.730	19.09
*rs109367522*	5	42871428	*PTPRR*	*rs42791351*	43371061	*LOC104972423*; *MYRFL*	−2.700	19.09
*rs110991306*	2	94592808	*GPR1*; *ZDBF2*	*rs110975979*	95586874	*LOC112442377*; *LOC100847666*	2.360	19.06
*rs43183410*	20	45022442	*LOC112443070* (u)	*rs137619389*	45663409	*LOC112443070*; *LOC112442955*	−3.230	19.02
*rs3423093320*	14	35595531	*LOC101903752*; *TRPA1*	*rs3423093914*	35815088	*LOC112449577*; *LOC112449516*	2.340	18.83

‘u’ indicates the SNP is upstream of the gene. ‘αα’ is the A × A effect of the SNP pair.

**Table 4:** Significant D × D effects.

SNP-1	Chr-1	Position-1	Candidate Gene-1	SNP-2	Chr-2	Position-2	Candidate Gene-2	δδ	log10(1/p)
*rs42670220*	20	39445232	*RAI14*	*rs41942366*	20	41041011	*LOC104975283*; *SUB1*	5.010	15.33
*rs110573919*	25	3828387	*DNAAF8*	*rs134957967*	25	3920360	*UBN1*	4.350	14.32
*rs109289243*	17	66235451	*TPST2*	*rs109255241*	17	66309374	*LOC100847159*; *LOC614881*	4.710	14.28
*rs132697751*	13	25821584	*ENKUR*	*rs109352819*	13	25911339	*GPR158*	4.750	14.25
*rs41942122*	20	34603769	*LOC782462*; *LOC112443045*	*rs109633897*	20	34759872	*LOC782462*; *LOC112443045*	5.230	13.94
*rs41874708*	18	32330449	*LOC112442399*; *CDH11*	*rs41637282*	18	32771279	*CDH11*	4.950	13.90
*rs132912044*	4	6268973	*LOC101904266* (d)	*rs3423164105*	4	6379484	*LOC101904266* (d)	4.600	13.87
*rs110405028*	18	14014113	*APRT*	*rs110180580*	18	14050260	*CBFA2T3*	4.500	13.48
*rs41594638*	7	75473623	*LOC100296952*; *LOC112447598*	*rs41667214*	7	76297401	*LOC112447528* (u)	4.250	13.29
*rs42640157*	20	58364680	*ANKH*	*rs110840725*	20	58566498	*OTULINL*	3.930	12.93
*rs109014148*	20	33024411	*PLCXD3*	*rs43001858*	20	33323766	*C6*	5.010	12.77
*rs43278453*	1	142383844	*UMODL1*	*rs136138690*	1	143444786	*CRYAA*; *LOC112448267*	4.130	12.72
*rs41951335*	20	58357512	*ANKH*	*rs110840725*	20	58566498	*OTULINL*	3.900	12.68
*rs134010835*	22	35456434	*MAGI1*	*rs110776523*	22	35535752	*MAGI1*	4.950	12.53
*rs41894282*	18	54627066	*BICRA*; *EHD2*	*rs3423454787*	18	54680124	*SELENOW*; *LOC101902766*	5.650	12.52
*rs135176671*	1	144551556	*TSPEAR*	*rs109840008*	1	146320854	*PRMT2*	4.030	12.37
*rs137324140*	13	2145874	*PLCB4*	*rs134403374*	13	2232129	*ENSBTAG00000054174*	4.470	12.14
*rs3423449147*	20	67492982	*LOC104969150*; *ICE1*	*rs41964197*	20	68697504	*LOC112443018* (d)	4.880	12.03

‘d’ indicates the SNP is downstream of the gene. ‘u’ indicates the SNP is upstream of the gene. ‘δδ’ is the D × D effect of the SNP pair.

**Table 5:** Daughter pregnancy rate (DPR) of crossbred, Holstein, and Jersey cows based on the CDCB first lactation DPR data of 2015–2023.

	Crossbred	Jersey	Holstein
Mean	54.9703 ± 33.178	53.434 ± 33.199	49.06 ± 32.01
n	31,338	722,949	4,956,093

## Data Availability

The original genotype data are owned by third parties and maintained by the Council on Dairy Cattle Breeding (CDCB). A request to the CDCB is necessary for obtaining data access on the research, which may be sent to: João Dürr, CDCB Chief Executive Officer (joao.durr@cdcb.us). All other relevant data are available in the manuscript and Supplementary Materials.

## Supplementary Materials

The following supporting information can be downloaded at: https://www.mdpi.com/article/10.3390/ijms262211149/s1.

## Appendix A. Candidate Genes with Fertility and Reproductive Functions

**SNP**	**Chr**	**Position**	**Candidate** **Gene**	**Effect Type**	**Function**
*rs110391667*	1	68682461	*KALRN*	AA^intra^	Embryo development [31], Holstein cow conception rate, and DPR [16]
*rs133450760*	1	68949628	*KALRN*	A	Embryo development [31], Holstein cow conception rate, and DPR [16]
*rs110468756*	1	75346472	*FGF12*	A	Progesterone synthesis [32]
*rs136701022*	1	75755758	*FGF12*	AA^intra^	Progesterone synthesis [32]
*rs110789800*	1	76375060	*GMNC*	A	Growth and fertility [33]
*rs41640538*	1	81200460	*ETV5*	A	Spermatogenesis and folliculogenesis [34,35]
*rs43278453*	1	142383844	*UMODL1*	DD^intra^	Oocyte quality [36]
*rs41583697*	1	146007587	*PCNT*	D	Female fertility [37]
*rs110783898*	1	153664085	*DAZL*	AA^intra^	Germ cells development in embryos [38]
*rs110991306*	2	94592808	*GPR1*	AA^intra^	Steroidogenesis in ovaries [39]
*rs110027085*	2	100793281	*ERBB4*	A	Regulation of sertoli and germ cell adhesion [40]
*rs43315219*	2	106815376	*WNT6*	AA^intra^	Stromal proliferation during decidualization [41]
*rs110099308*	2	122454418	*PUM1*	D	Establishment of the primordial follicle pool [42]
*rs134335964*	2	128057688	*RUNX3*	AA^intra^	Regulates ovarian functions and ovulation [43]
*rs110808151*	3	19209742	*TUFT1*	AA^intra^	Male fertility [44]
*rs111002920*	3	27938935	*NGF*	AA^intra^	Folliculogenesis and spermatogenesis [45,46]
*rs43712201*	3	97857026	*SPATA6*	A	Essential for sperm formation [47]
*BTB-01885061*	4	38854540	*HGF*	A	Male and female fertility [48]
*rs43406699*	4	76069283	*IGFBP3*	AA^intra^	Follicle and pregnancy maintenance [49,50]
*rs42664831*	4	77659486	*HECW1*	AA^intra^	Oogenesis [51]
*rs110544309*	4	77811545	*HECW1*	DA^inter^	Oogenesis [51]
*BTB-01477571*	5	43254214	*CNOT2*	A	Oocyte maturation and female fertility [52]
*rs43438147*	5	43898744	*FRS2*	A	Epididymal development [53]
*rs136525396*	5	45641750	*IFNG*	AA^intra^	Success of fertilization and embryo development [54]
*rs134371136*	5	47933174	*HMGA2*	A	Male and female infertility [55]
*rs42516642*	5	75694606	*RAC2*	A	Remodeling of the endometrial stroma [56]
*rs110601510*	6	70246689	*KIT*	A	Male infertility [57]
*rs41654346*	6	72581115	*IGFBP7*	A, D	Maintenance of pregnancy [58]
*rs134948894*	6	81156919	*EPHA5*	AA^intra^	Female fertility [59]
*rs3423226650*	6	85656834	*CSN3*	A	Holstein DPR [16]
*rs43474151*	6	86508590	*SLC4A4*	A	Holstein DPR [16]
*rs110434046*	6	87184768	*GC*	A	Holstein cow conception rate and DPR [16]
*rs109034709*	6	87316810	*NPFFR2*	A	Holstein cow conception rate and DPR [16]
*rs43475934*	6	92430962	*CNOT6L*	A	Female fertility [60]
*rs110103910*	7	52664571	*DIAPH1*	AA^inter^	Sex hormone production [61]
*rs136410320*	7	61491583	*PDGFRB*	AA^intra^	Embryonic testis development [62]
*rs43549012*	8	36818848	*PTPRD*	AA^inter^	Gestational diabetes risk in pregnant women [63]
*rs109347873*	8	82976354	*HSD17B3*	A	Ovary morphological traits of cows [64]
*rs135050343*	8	105307551	*PAPPA*	A	Ovarian steroidogenesis and female fertility [65]
*UA-IFASA-5710*	9	15743940	*IMPG1*	D	Male fertility [66]
*rs29019953*	10	10520078	*HOMER1*	AA^intra^	Gonadotropin [67]
*rs134257923*	10	79265033	*EIF2S1*	D	Placentation and pregnancy maintenance [68]
*rs110147881*	12	20815304	*SERPINE3*	D	Follicle count and embryo development [69]
*rs42391346*	13	1641651	*PCLB1*	AA^intra^	Embryonic implantation failure [70]
*rs108997231*	13	3906114	*SLX4IP*	A	DNA repair and maintenance [71] and embryogenesis [18]
*rs41605729*	13	3710010	*SLX4IP*	AA^intra^	DNA repair and maintenance [71] and embryogenesis [18]
*rs132697751*	13	25821584	*ENKUR*	DD^intra^	Sperm motility [72]
*rs41704549*	13	58979512	*BMP7*	AA^intra^	Embryo development [73]
*rs41587252*	13	76013752	*NCOA3*	A	Germ cell and fertility-related phenotypes [74]
*rs3423093320*	14	35595531	*TRPA1*	AA^intra^	Spermatogenesis and uterine function [75,76]
*rs134052004*	14	40390594	*PEX2*	A, AA^intra^	Male fertility [77]
*rs110600324*	15	75950150	*PEX16*	A	Spermatocyte maturation [78]
*rs41576959*	16	15915181	*BRINP3*	D	Influencing horse reproductive traits [79]
*rs110348666*	17	25067804	*PCDH10*	AA^intra^	Embryo development [80]
*rs109778958*	17	29595964	*LARP1B*	A	Oogenesis [81]
*rs109289243*	17	66235451	*TPST2*	DD^intra^	Male infertility [82]
*rs136993800*	18	12377354	*FOXC2*	AA^intra^	Embryo development [83]
*rs109688013*	18	14705671	*MC1R*	D	Fertility and prenatal development [84]
*rs137810605*	18	16507273	*ABCC12*	AA^inter^	Male fertility [85]
*rs135072252*	18	40169055	*HYDIN*	AA^intra^	Sperm-tail movement [86]
*rs133429914*	18	43836519	*PEPD*	A	Holstein cow conception rate and DPR [16]
*rs41879557*	18	44031531	*CHST8*	A	Holstein cow conception rate, DPR [16], and productive life [87]
*rs110061700*	18	47814171	*SIPA1L3*	A	Embryo development [31], Holstein cow conception rate, and DPR [16]
*rs133706373*	18	56085584	*PRMT1*	D	Male fertility and embryo development [88,89]
*rs41600357*	19	58869903	*SOX9*	AA^intra^	Testis development and fertility [90]
*rs110823845*	19	61080519	*MAP2K6*	A	Testis determination [91]
*rs41638404*	20	25229625	*NDUFS4*	A	Embryo development [92]
*rs42278649*	20	29647234	*HCN1*	AD^intra^	Functional significance in ovaries [93]
*rs135853296*	20	32077537	*GHR*	A	Ovarian follicular growth [94]
*rs109014148*	20	33024411	*PLCXD3*	DD^intra^	Spermatogenic dysfunction [95]
*rs41257066*	20	35956386	*LIFR*	A	Embryo implantation [96]
*rs41944664*	20	41658534	*PDZD2*	D	Risk factor to recurrent miscarriage [97]
*rs110116785*	20	41999241	*DROSHA*	D	Embryo development [98]
*rs109535019*	20	46964318	*CDH9*	AD^intra^	Sperm capacitation and egg—sperm binding [99]
*rs42986448*	20	51571771	*CDH12*	AA^inter^	Female fertility [100]
*rs109901314*	21	59873899	*DICER1*	AA^intra^	Female fertility [101]
*rs41993852*	22	1986320	*EOMES*	A	Embryo development [102]
*rs109972368*	22	17708467	*OXTR*	AA^intra^	Uterine contraction and pregnancy maintenance [103]
*rs137590982*	22	17910318	*LMCD1*	A	Male fertility [104]
*rs136164685*	22	17954298	*LMCD1*	AA^intra^	Male fertility [104]
*rs137797676*	23	9112456	*TCP11*	AA^intra^	Sperm capacitation, motility, and acrosome reaction [105,106]
*rs110080847*	24	21456850	*INO80C*	A	Spermatogenesis [107]
*rs42052893*	24	61612123	*KDSR*	A	Embryo development [108]
*rs110028007*	27	40780768	*RARB*	AA^intra^	Embryo development [109]
*rs134769462*	27	41433477	*THRB*	A, D	Female fertility [110]
*rs133507315*	29	11356071	*DLG2*	A, AA^intra^	Delayed puberty [111]
In ‘Effect Type’ column, A = additive effect, D = dominance effect, AA^intra^ = intra-chromosome A × A effect, AA^inter^ = inter-chromosome A × A effect, AD^intra^ = intra-chromosome A × D effect, AD^inter^ = inter-chromosome A × D effect, and DD^intra^ = intra-chromosome D × D effect, DA^inter^ = inter-chromosome D × A effect.					
